# Implementation of the e-SUS Primary Care Strategy: an analysis based on official data

**DOI:** 10.11606/s1518-8787.2022056003405

**Published:** 2022-02-23

**Authors:** Ana Claudia Cielo, Tainá Raiol, Everton Nunes da Silva, Jorge Otávio Maia Barreto

**Affiliations:** I Fundação Oswaldo Cruz Programa de Mestrado Profissional em Políticas Públicas em Saúde Brasília DF Brasil Fundação Oswaldo Cruz. Programa de Mestrado Profissional em Políticas Públicas em Saúde. Brasília, DF, Brasil; II Universidade de Brasília Faculdade de Ceilândia Brasília DF Brasil Universidade de Brasília. Faculdade de Ceilândia. Brasília, DF, Brasil

**Keywords:** Unified Health System, Electronic Health Records, Health Information Systems, Primary Health Care, eHealth Strategies

## Abstract

**OBJETIVE:**

Analyze the implementation of the strategy *e-SUS Atenção Básica* (e-SUS AB – e-SUS Primary Care) in Brazil between the first years of the system, from 2013 to 2019.

**METHODS:**

This is a quantitative, descriptive, and exploratory study. We considered official data from the Ministry of Health, submitted by Brazilian municipalities, in the period from April 2013 to December 2019. We categorized the municipalities as ‘not implemented’, ‘initial implementation’, ‘partial implementation’ and ‘implemented’ according to the criteria defined in this study. We also verified whether the type of municipality, according to the IBGE classification, influenced the degree of implementation of the e-SUS AB strategy. We performed descriptive analyses and investigated the association between the degrees of implementation of e-SUS AB and the typology of the IBGE classification and characterization of rural and urban spaces.

**RESULTS:**

The implementation increased in the analyzed period. The implementation status of the e-SUS AB strategy in 2019 was ‘implemented’ in 20.2% (1,117) of the municipalities, ‘partial implementation’ in 32.9% (1,819), ‘initial implementation’ in 39.1% (2,159) and ‘not implemented’ in 7.8% (432). The South and Southeast regions presented the best implementation situation in all years, and the states of Rio Grande do Sul, São Paulo and Santa Catarina reached a higher percentage of municipalities with ‘implemented’ status in 2019.

**CONCLUSIONS:**

We confirmed the progress in the implementation of the e-SUS AB strategy over the years. Most of the municipalities are between the status ‘initial implementation’ and ‘partial implementation’. Therefore, we conclude that investments in technological resources, training of professionals, and support are necessary to qualify the implementation and use of information systems in the country, especially for the e-SUS AB strategy.

## INTRODUCTION

Health information systems (HIS) are standardized data collection and monitoring tools designed to provide information for health analysis, aiming at improving the understanding of the population’s health problems^1,2^, subsidizing decision making in public policies^3^.

Historically, Brazilian HIS are fragmented. With multiple sources, the collected data is consolidated with low quality and its availability adopts a format that hinders its appropriation and use by health managers^4,5^. Thus, the monitoring of the quality of the data that serves the Brazilian Unified Health System (SUS) does not follow a regular plan of evaluations, with only isolated initiatives^6^.

The Brazilian government, inspired by successful experiences in countries in Europe, in Canada, Australia and New Zealand, among others, conceived and adopted the e-Health Strategy for Brazil. The e-Health Strategy aims to increase the quality and expand access to health care, qualify the teams, streamline care and improve the flow of information for clinical decision making, surveillance, regulation and health promotion. In addition, it aims at decision making focused on health management^7^, in line with the National Information and Informatics Policy, which, in its latest version in 2016, reinforced the importance of guiding information and communication technology (ICT) actions and standardizing the collection and processing of health system data^8^.

In primary care (PC), the strategy *e-SUS Atenção Básica* (e-SUS AB – e-SUS Primary Care) was created in 2013^9,10^. It proposed to offer a new health information system to meet the different informatization and organization needs of the municipalities. Its goal was to modernize the technological platform, supporting care management, optimizing data collection, interfacing with the various systems used by primary care, and improving the detailing of health information^11^. This would be possible through the National Health Card, which allows the individualization of records, which was a great challenge for Brazil, because it broke the logic of consolidated data used in primary care until then.

Three years after the system was made available, it became mandatory to send information to the database of the *Sistema de Informação em Saúde para a Atenção Básica* (SISAB – Health Information System for Primary Care)^10^.

The e-SUS AB strategy includes the national information repository SISAB and two collection software for entering primary data recorded by primary care professionals: 1) *Coleta de Dados Simplificada* (CDS – Simplified Data Collection), using paper forms; and 2) *Prontuário Eletrônico do Cidadão* (PEC – Electronic Citizen Record), a computational system. It also includes the systems sold by third parties or the municipalities’ own systems, integrated by means of a previously defined data import mechanism ^1^.

The movement generated by the change in the information system impelled the informatization of primary care services throughout the country and can be considered a success story among the available systems at the federal level. Souza et al.^10^ highlight that, in 2018, the e-SUS AB strategy was present in the 5,562 Brazilian municipalities with primary care services and around 98% of the family health teams (eSF– *Saúde da Família*), representing more than 42.8 thousand teams.

Despite the visible progress, studies analyzing the evolution of the e-SUS AB implementation are still scarce, as well as the factors that influence this process and the time needed to implement the strategy. It is plausible the existence of different degrees of implementation of the strategy, influenced by the characteristics of the municipalities, such as their location, population density, urbanization, level of informatization, etc. Geographic and socioeconomic factors, in general, are relevant for government initiatives that require the prior availability of specific resources for their implementation, while they are measurable and available, in official databases, facilitating their use for monitoring the progress of implementation.

This study analyzed the implementation of the e-SUS AB strategy in Brazil in the period from 2013 to 2019, also investigating municipal characteristics that potentially influenced the best performance in this process.

## METHODS

### Study Design

This is a quantitative, descriptive and exploratory study, based on administrative data. We estimated the degree of implementation of e-SUS AB at the municipal level, considering the period from April 2013 to December 2019. Thus, the study population consists of the Brazilian municipalities offering Primary Care services registered in the *Cadastro Nacional de Estabelecimentos de Saúde* (CNES – National Registry of Health Care Establishments). The number of municipalities presented a variation in the years studied: 5,454 (2013); 5,496 (2014); 5,514 (2015); 5,517 (2016); 5,522 (2017); 5,524 (2018); and 5,527 (2019). We based the stage of implementation in each municipality on data provided by the primary care/family health teams to the Ministry of Health regarding the use of e-SUS AB. We excluded from this study *Atenção Básica Prisional* (EABP – Prison Primary Care Teams), *Núcleo de Apoio a Saúde da Família* (NASF – Family Health Support Center), *Consultório na Rua* (CnR – Street Clinic), isolated Oral Health Teams (not linked to an ESF), and Basic Health Units with no linked teams.

### Procedures Adopted to Estimate the Percentage of e-SUS Implementation

The variables used in this study reflect the systematic sending of data to SISAB: competence (month), Federated Unit (FU), municipality code from the Brazilian Institute of Geography and Statistics (IBGE), health unit code from CNES, team code from the *Identificador Nacional de Equipes* (INE – National Team Identifier), team type code from CNES, professional category, and valid records in the CNES national base.

The preparation of the database preceded the data analysis, comprising the following processing steps: identification of the active professional categories in the CNES per month; verification of the information sent to SISAB by the active professional categories in the CNES (doctor, nurse, nursing technician and assistant, dentist, oral health technician and assistant, and community health agent); analysis of the information sent to SISAB by Primary Care/Family Health team. In this step, we analyzed whether the professional categories of each team sent information, classifying the teams between those that sent information and those that did not, according to the criteria defined for the study.

In the next step, we defined and applied the criteria to characterize the degree of implementation of e-SUS AB, considering the frequency and regularity of data submission to SISAB. First, we considered data submission by team: a) Insufficient Submission – no submission of information or submission of less than 30% of the year’s competencies; b) Initial Submission – Submission of information above 30% of the year’s competencies or submission of information for three (3) consecutive competencies in the year; c) Partial Submission – Submission of information above 50% of the year’s competencies and submission of information for three (3) consecutive competencies in the year; d) Satisfactory Submission – Submission of information above 80% of the year’s competencies and three (3) consecutive competencies in the year.

**Figure 1:**
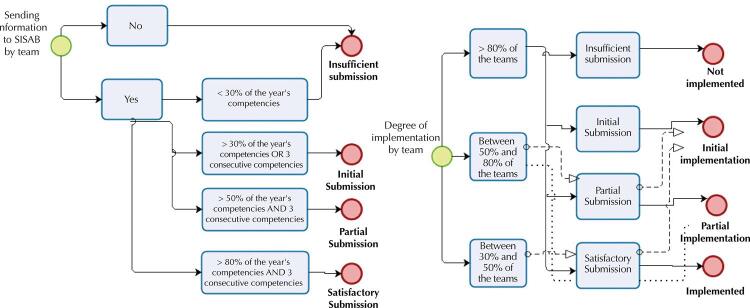
Flowchart of the analysis of the implementation of the e-SUS AB Strategy.

Next, the results of the previous step were used to assess the degree of implementation of the e-SUS AB strategy in the municipalities: a) Not Implemented – more than 80% of the teams in the municipality classified as ‘insufficient submission’ in the previous phase; b) Initial Implementation – more than 80% of the teams in the municipality classified as ‘initial submission’ or between 50% and 80% of the teams classified as ‘partial submission’ or between 30% and 50% of the teams classified as ‘satisfactory submission’ in the previous phase; c) Partial Implementation – more than 80% of the municipality’s teams classified as ‘partial submission’ or between 50% and 80% of the teams classified as ‘satisfactory submission’ in the previous phase; d) Implemented – more than 80% of the municipality’s teams classified as ‘satisfactory submission’ in the previous phase. Figure 1 shows the flow of the classification performed.

### Statistical Analysis

The data was calculated using the ‘team’ as the unit of analysis, and then aggregated by municipality, and finally by state, by geographic region, and nationally. We presented the data as maps and graphs and made the analysis using the programming language *R* and *RStudio* (version 1.1.463, 2009 – 2018^12^, package plyr, dplyr, readxl and rlist).

Finally, we also used the IBGE^13^ classification for characterizing rural and urban spaces to investigate the implementation of e-SUS AB, considering the groups defined in it. This classification considers aspects related to population density and its distribution in the territory of the municipalities, to define five groups, applicable to the characterization of Brazilian municipalities: urban, adjacent intermediate, remote intermediate, adjacent rural, and remote rural.

### Ethical Considerations

We conducted the research in accordance with ethical research standards. This study used secondary and unidentified data from official information systems of the Ministry of Health, exempted from ethics committee review, as provided in Resolution No. 510, April 7, 2016^14^.

## RESULTS

**Figure 2:**
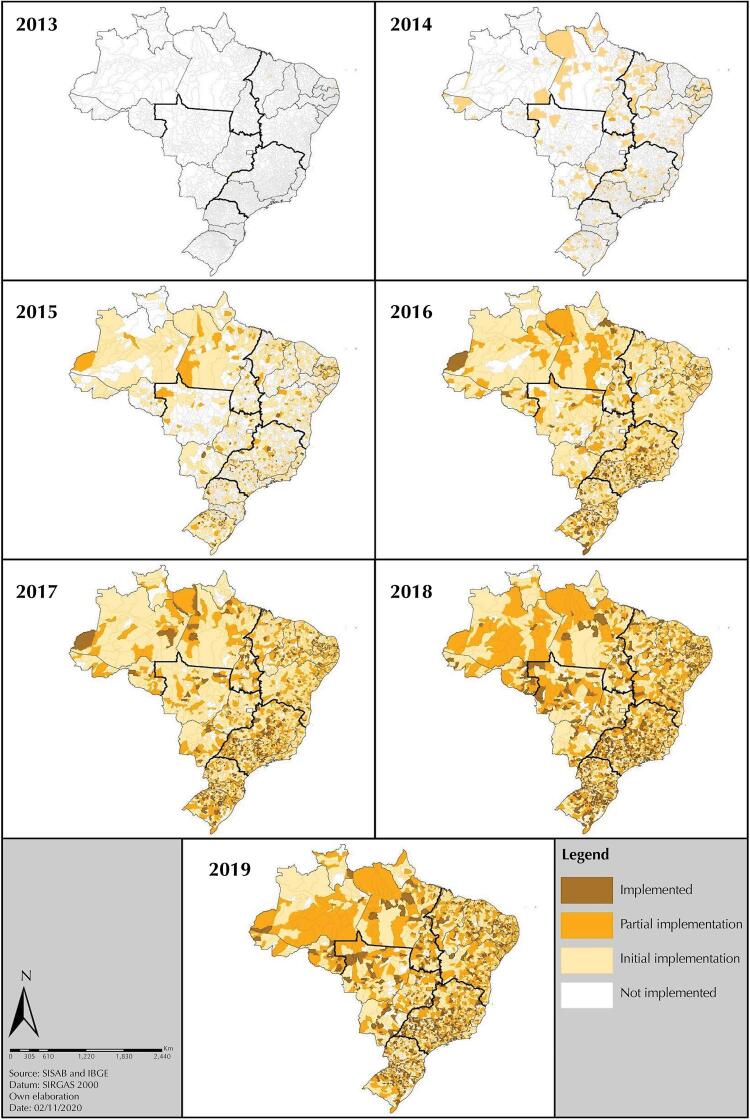
Evolution of the implementation status of the e-SUS AB Strategy by municipality, Brazil, from 2013 to 2019.

The aggregated national scale results of the implementation of the e-SUS AB strategy show expressive growth in the implementation of the system. The Figure 2 shows the evolution of the implementation of this strategy in Brazil from 2013 to 2019. In 2013 and 2014, the classification of the status of municipalities were 99.7% and 83.4% to ‘not implemented’, respectively. In 2015, 49.2% of municipalities achieved some degree of implementation other than ‘not implemented’ status. In 2016 and 2017, the ‘initial implementation’ and ‘partial implementation’ statuses exceeded the ‘not implemented’ percentage and the largest share was between the ‘initial implementation’ status, 49.7% and 48.3%, and ‘partial implementation’ status, 21.6% and 28.7%, respectively. In the last two years of analysis, 2018 and 2019, 37.9% and 39.1% of municipalities had ‘initial implementation’ status; 32.4% and 32.9%, ‘partial implementation’; and with ‘implemented’ status, 21.7% and 20.2%, respectively.

**Figure 3:**
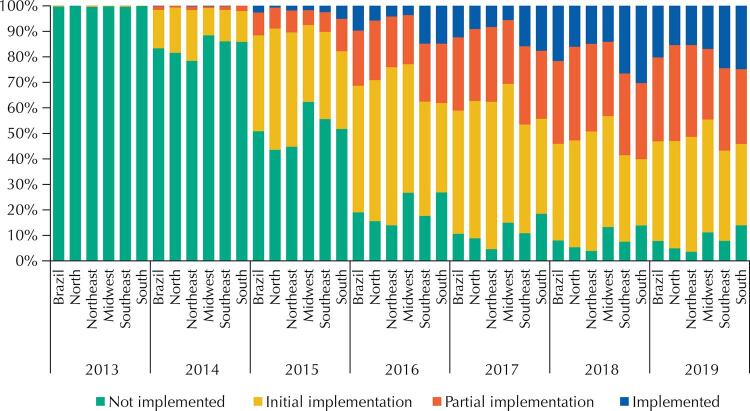
Status of implementation of the e-SUS AB Strategy by geographic region, Brazil, from 2013 to 2019.

The evolution of the implementation of the e-SUS AB strategy happened differently among the country’s regions, as shown in Figure 3. In terms of the percentage of implementation, the North, Northeast, and Midwest regions were below the national average in every year since 2015, while the Southeast and South regions were above the national average in the same period. In 2019, the percentages of municipalities in the ‘implemented’ and ‘partial implementation’ situation exceed the ‘not implemented’ and ‘initial implementation’ situation in Brazil. In this aspect, the Southeast region presents the best situation, with 56.8% of the municipalities in this situation; followed by the South region, 54.1%; the North, 53%; the Northeast, 51.4%; and the Midwest, 44.5%. The analysis of the best percentage of municipalities with ‘implemented’ status highlights the South region (24.8%); followed by the Southeast region (24.4%); the Midwest (16.9%); and the Northeast and North (15.4%) with the same percentage.

In the analysis of implementation by State (Table 1), we observed that the highest percentage of municipalities with the ‘implemented’ situation in 2019 was in the state of Rio Grande do Sul (30.3%), followed by São Paulo (29.5%) and Santa Catarina (28.8%). The state with the most deficient situation is Amapá, with 4.5% of the municipalities with the ‘implemented’ status. Distrito Federal, despite its peculiarities, did not present the ‘implemented’ situation. The Brazilian states with the highest percentage of municipalities with the ‘not implemented’ situation were Roraima with 20%, Paraná with 15.3%, Rio Grande do Sul, and Goiás with 13.7%. Alagoas also had no municipality with a ‘not implemented’ situation.

**Table:** Implementation of the e-SUS AB Strategy by state and region, 2013 to 2019.

Region/FU	Status of implementation	2013 n (%)	2014 n (%)	2015 n (%)	2016 n (%)	2017 n (%)	2018 n (%)	2019 n (%)
North								
AC	Not implemented	22 (100)	18 (81.8)	10 (45.4)	6 (27.2)	3 (13.6)	0 (0)	2 (9)
	Initial implementation	0 (0)	4 (18.1)	12 (54.5)	12 (54.5)	12 (54.5)	14 (63.6)	14 (63.6)
	Partial implementation	0 (0)	0 (0)	0 (0)	4 (18.1)	7 (31.8)	7 (31.8)	5 (22.7)
	Implemented	0 (0)	0 (0)	0 (0)	0 (0)	0 (0)	1 (4.5)	1 (4.5)
AM	Not implemented	62 (100)	57 (91.9)	18 (29)	4 (6.4)	2 (3.2)	1 (1.6)	1 (1.6)
	Initial implementation	0 (0)	5 (8)	41 (66.1)	43 (69.3)	41 (66.1)	30 (48.3)	30 (48.3)
	Partial implementation	0 (0)	0 (0)	3 (4.8)	14 (22.5)	16 (25.8)	28 (45.1)	27 (43.5)
	Implemented	0 (0)	0 (0)	0 (0)	1 (1.6)	3 (4.8)	3 (4.8)	4 (6.4)
AP	Not implemented	16 (100)	13 (81.2)	7 (43.7)	8 (50)	6 (37.5)	3 (18.7)	1 (6.2)
	Initial implementation	0 (0)	3 (18.7)	8 (50)	7 (43.7)	9 (56.2)	10 (62.5)	13 (81.2)
	Partial implementation	0 (0)	0 (0)	1 (6.2)	1 (6.2)	1 (6.2)	3 (18.7)	2 (12.5)
PA	Not implemented	143 (100)	107 (74.3)	39 (27)	7 (4.8)	3 (2)	2 (1.3)	1 (0.6)
	Initial implementation	0 (0)	37 (25.6)	84 (58.3)	91 (63.1)	95 (65.9)	74 (51.3)	73 (50.6)
	Partial implementation	0 (0)	0 (0)	20 (13.8)	38 (26.3)	34 (23.6)	54 (37.5)	57 (39.5)
	Implemented	0 (0)	0 (0)	1 (0.6)	8 (5.5)	12 (8.3)	14 (9.7)	13 (9)
RO	Not implemented	52 (100)	47 (90.3)	26 (50)	6 (11.5)	3 (5.7)	2 (3.8)	1 (1.9)
	Initial implementation	0 (0)	5 (9.6)	23 (44.2)	30 (57.6)	27 (51.9)	17 (32.6)	19 (36.5)
	Partial implementation	0 (0)	0 (0)	3 (5.7)	13 (25)	18 (34.6)	24 (46.1)	19 (36.5)
	Implemented	0 (0)	0 (0)	0 (0)	3 (5.7)	4 (7.6)	9 (17.3)	13 (25)
RR	Not implemented	15 (100)	14 (93.3)	12 (80)	3 (20)	4 (26.6)	1 (6.6)	3 (20)
	Initial implementation	0 (0)	1 (6.6)	3 (20)	12 (80)	8 (53.3)	10 (66.6)	8 (53.3)
	Partial implementation	0 (0)	0 (0)	0 (0)	0 (0)	3 (20)	4 (26.6)	4 (26.6)
TO	Not implemented	139 (100)	111 (79.8)	84 (60.4)	36 (25.8)	19 (13.6)	15 (10.8)	13 (9.4)
	Initial implementation	0 (0)	25 (17.9)	43 (30.9)	54 (38.8)	50 (35.9)	33 (23.9)	32 (23.1)
	Partial implementation	0 (0)	3 (2.1)	10 (7.1)	35 (25.1)	48 (34.5)	45 (32.6)	55 (39.8)
	Implemented	0 (0)	0 (0)	2 (1.4)	14 (10)	22 (15.8)	45 (32.6)	38 (27.5)
Northeast								
AL	Not implemented	102 (100)	98 (96)	58 (56.8)	2 (1.9)	0 (0)	0 (0)	0 (0)
	Initial implementation	0 (0)	4 (3.9)	40 (39.2)	59 (57.8)	51 (50)	35 (34.3)	27 (26.4)
	Partial implementation	0 (0)	0 (0)	4 (3.9)	36 (35.2)	36 (35.2)	41 (40.1)	48 (47)
	Implemented	0 (0)	0 (0)	0 (0)	5 (4.9)	15 (14.7)	26 (25.4)	27 (26.4)
BA	Not implemented	417 (100)	344 (82.4)	200 (47.9)	47 (11.2)	11 (2.6)	12 (2.8)	5 (1.1)
	Initial implementation	0 (0)	72 (17.2)	187 (44.8)	297 (71.2)	267 (64)	190 (45.5)	190 (45.5)
	Partial implementation	0 (0)	1 (0.2)	25 (5.9)	65 (15.5)	113 (27)	163 (39)	158 (37.8)
	Implemented	0 (0)	0 (0)	5 (1.1)	8 (1.9)	26 (6.2)	52 (12.4)	64 (15.3)
CE	Not implemented	184 (100)	183 (99.4)	92 (50)	22 (11.9)	7 (3.8)	7 (3.8)	5 (2.7)
	Initial implementation	0 (0)	1 (0.5)	88 (47.8)	135 (73.3)	126 (68.4)	98 (53.2)	91 (49.4)
	Partial implementation	0 (0)	0 (0)	4 (2.1)	21 (11.4)	36 (19.5)	62 (33.6)	66 (35.8)
	Implemented	0 (0)	0 (0)	0 (0)	6 (3.2)	15 (8.1)	17 (9.2)	22 (11.9)
MA	Not implemented	216 (99.5)	140 (64.5)	82 (37.7)	40 (18.4)	16 (7.3)	11 (5)	13 (5.9)
	Initial implementation	1 (0.4)	74 (34.1)	118 (54.3)	138 (63.5)	149 (68.6)	125 (57.6)	119 (54.8)
	Partial implementation	0 (0)	3 (1.3)	14 (6.4)	35 (16.1)	41 (18.8)	56 (25.8)	61 (28.1)
	Implemented	0 (0)	0 (0)	3 (1.3)	4 (1.8)	11 (5)	25 (11.5)	24 (11)
PB	Not implemented	220 (98.6)	114 (51.1)	43 (19.2)	22 (9.8)	10 (4.4)	10 (4.4)	10 (4.4)
	Initial implementation	2 (0.8)	97 (43.4)	104 (46.6)	102 (45.7)	95 (42.6)	87 (39)	92 (41.2)
	Partial implementation	1 (0.4)	9 (4)	58 (26)	73 (32.7)	85 (38.1)	75 (33.6)	84 (37.6)
	Implemented	0 (0)	3 (1.3)	18 (8)	26 (11.6)	33 (14.7)	51 (22.8)	37 (16.5)
PE	Not implemented	185 (100)	161 (87)	93 (50.2)	15 (8.1)	3 (1.6)	1 (0.5)	2 (1)
	Initial implementation	0 (0)	23 (12.4)	83 (44.8)	131 (70.8)	116 (62.7)	94 (50.8)	86 (46.4)
	Partial implementation	0 (0)	1 (0.5)	8 (4.3)	32 (17.2)	56 (30.2)	61 (32.9)	74 (40)
	Implemented	0 (0)	0 (0)	1 (0.5)	7 (3.7)	10 (5.4)	29 (15.6)	23 (12.4)
PI	Not implemented	224 (100)	212 (94.6)	138 (61.6)	62 (27.6)	24 (10.7)	18 (8)	20 (8.9)
	Initial implementation	0 (0)	11 (4.9)	74 (33)	110 (49.1)	114 (50.8)	103 (45.9)	100 (44.6)
	Partial implementation	0 (0)	1 (0.4)	10 (4.4)	45 (20)	70 (31.2)	70 (31.2)	71 (31.6)
	Implemented	0 (0)	0 (0)	2 (0.8)	7 (3.1)	16 (7.1)	33 (14.7)	33 (14.7)
RN	Not implemented	163 (97.6)	110 (65.8)	66 (39.5)	30 (17.9)	11 (6.5)	7 (4.1)	5 (2.9)
	Initial implementation	4 (2.3)	48 (28.7)	75 (44.9)	93 (55.6)	82 (49.1)	81 (48.5)	76 (45.5)
	Partial implementation	0 (0)	9 (5.3)	25 (14.9)	37 (22.1)	56 (33.5)	56 (33.5)	55 (32.9)
	Implemented	0 (0)	0 (0)	1 (0.5)	7 (4.1)	18 (10.7)	23 (13.7)	31 (18.5)
SE	Not implemented	75 (100)	45 (60)	31 (41.3)	10 (13.3)	1 (1.3)	4 (5.3)	4 (5.3)
	Initial implementation	0 (0)	28 (37.3)	35 (46.6)	47 (62.6)	36 (48)	28 (37.3)	27 (36)
	Partial implementation	0 (0)	2 (2.6)	7 (9.3)	13 (17.3)	33 (44)	32 (42.6)	29 (38.6)
	Implemented	0 (0)	0 (0)	2 (2.6)	5 (6.6)	5 (6.6)	11 (14.6)	15 (20)
Midwest								
DF	Not implemented	1 (100)	1 (100)	1 (100)	1 (100)	1 (100)	0 (0)	0 (0)
	Initial implementation	0 (0)	0 (0)	0 (0)	0 (0)	0 (0)	1 (100)	0 (0)
	Partial implementation	0 (0)	0 (0)	0 (0)	0 (0)	0 (0)	0 (0)	1 (100)
	Implemented	0 (0)	0 (0)	0 (0)	0 (0)	0 (0)	0 (0)	0 (0)
GO	Not implemented	245 (99.5)	219 (89)	144 (58.5)	73 (29.6)	42 (17)	39 (15.8)	33 (13.4)
	Initial implementation	1 (0.4)	25 (10.1)	78 (31.7)	113 (45.9)	126 (51.2)	108 (43.9)	105 (42.6)
	Partial implementation	0 (0)	1 (0.4)	17 (6.9)	50 (20.3)	64 (26)	65 (26.4)	70 (28.4)
	Implemented	0 (0)	1 (0.4)	7 (2.8)	10 (4)	14 (5.6)	34 (13.8)	38 (15.4)
MS	Not implemented	79 (100)	63 (79.7)	44 (55.6)	15 (18.9)	6 (7.5)	5 (6.3)	3 (3.7)
	Initial implementation	0 (0)	15 (18.9)	31 (39.2)	47 (59.4)	52 (65.8)	43 (54.4)	46 (58.2)
	Partial implementation	0 (0)	1 (1.2)	3 (3.7)	15 (18.9)	17 (21.5)	23 (29.1)	16 (20.2)
	Implemented	0 (0)	0 (0)	1 (1.2)	2 (2.5)	4 (5)	8 (10.1)	14 (17.7)
MT	Not implemented	141 (100)	130 (92.1)	102 (72.3)	36 (25.5)	21 (14.8)	18 (12.7)	16 (11.3)
	Initial implementation	0 (0)	10 (7)	32 (22.6)	75 (53.1)	76 (53.9)	51 (36.1)	56 (39.7)
	Partial implementation	0 (0)	1 (0.7)	7 (4.9)	25 (17.7)	36 (25.5)	48 (34)	42 (29.7)
	Implemented	0 (0)	0 (0)	0 (0)	5 (3.5)	8 (5.6)	24 (17)	27 (19.1)
Southeast								
ES	Not implemented	78 (100)	70 (89.7)	33 (42.3)	14 (17.9)	9 (11.5)	10 (12.8)	8 (10.2)
	Initial implementation	0 (0)	7 (8.9)	39 (50)	47 (60.2)	51 (65.3)	42 (53.8)	48 (61.5)
	Partial implementation	0 (0)	1 (1.2)	3 (3.8)	14 (17.9)	16 (20.5)	21 (26.9)	17 (21.7)
	Implemented	0 (0)	0 (0)	3 (3.8)	3 (3.8)	2 (2.5)	5 (6.4)	5 (6.4)
MG	Not implemented	850 (99.8)	741 (86.9)	486 (57)	128 (15)	76 (8.9)	59 (6.9)	63 (7.3)
	Initial implementation	1 (0.1)	100 (11.7)	285 (33.4)	382 (44.8)	360 (42.2)	288 (33.7)	287 (33.6)
	Partial implementation	0 (0)	11 (1.2)	70 (8.2)	210 (24.6)	275 (32.2)	280 (32.8)	295 (34.5)
	Implemented	0 (0)	0 (0)	11 (1.2)	132 (15.4)	142 (16.6)	226 (26.4)	208 (24.3)
RJ	Not implemented	91 (100)	68 (74.7)	36 (39.1)	5 (5.4)	7 (7.6)	3 (3.2)	7 (7.6)
	Initial implementation	0 (0)	22 (24.1)	50 (54.3)	76 (82.6)	67 (72.8)	57 (61.9)	56 (60.8)
	Partial implementation	0 (0)	1 (1)	5 (5.4)	8 (8.6)	16 (17.3)	25 (27.1)	23 (25)
	Implemented	0 (0)	0 (0)	1 (1)	3 (3.2)	2 (2.1)	7 (7.6)	6 (6.5)
SP	Not implemented	567 (99.1)	512 (86)	349 (57.7)	140 (23)	85 (13.9)	51 (8.3)	51 (8.3)
	Initial implementation	4 (0.6)	71 (11.9)	182 (30.1)	226 (37.2)	218 (35.7)	167 (27.3)	187 (30.5)
	Partial implementation	1 (0.1)	10 (1.6)	48 (7.9)	137 (22.5)	193 (31.6)	197 (32.2)	194 (31.6)
	Implemented	0 (0)	2 (0.3)	25 (4.1)	104 (17.1)	113 (18.5)	195 (31.9)	181 (29.5)
South								
PR	Not implemented	398 (100)	360 (90.2)	231 (57.8)	144 (36)	80 (20)	55 (13.8)	61 (15.3)
	Initial implementation	0 (0)	37 (9.2)	134 (33.5)	166 (41.6)	200 (50.1)	135 (33.9)	170 (42.7)
	Partial implementation	0 (0)	2 (0.5)	28 (7)	73 (18.2)	85 (21.3)	122 (30.6)	107 (26.8)
	Implemented	0 (0)	0 (0)	6 (1.5)	16 (4)	34 (8.5)	86 (21.6)	60 (15)
RS	Not implemented	459 (99.7)	360 (75.7)	175 (36.2)	103 (21.3)	94 (19.3)	73 (14.9)	67 (13.7)
	Initial implementation	1 (0.2)	93 (19.5)	149 (30.8)	130 (26.9)	125 (25.7)	97 (19.8)	120 (24.5)
	Partial implementation	0 (0)	22 (4.6)	105 (21.7)	135 (27.9)	146 (30.1)	139 (28.4)	153 (31.3)
	Implemented	0 (0)	0 (0)	54 (11.1)	115 (23.8)	120 (24.7)	179 (36.6)	148 (30.3)
SC	Not implemented	294 (100)	284 (96.2)	203 (68.8)	69 (23.3)	44 (14.9)	36 (12.2)	37 (12.5)
	Initial implementation	0 (0)	11 (3.7)	75 (25.4)	117 (39.6)	114 (38.6)	75 (25.4)	87 (29.4)
	Partial implementation	0 (0)	0 (0)	17 (5.7)	65 (22)	83 (28.1)	91 (30.8)	86 (29.1)
	Implemented	0 (0)	0 (0)	0 (0)	44 (14.9)	54 (18.3)	93 (31.5)	85 (28.8)
Overall Total		5,454 (100)	5,496 (100)	5,514 (100)	5,517 (100)	5,522 (100)	5,524 (100)	5,527 (100)

FU: AC: Acre; AL: Alagoas; AP: Amapá; AM: Amazonas; BA: Bahia; CE: Ceará; DF: Distrito Federal; ES: Espírito Santo; GO: Goiás; MA: Maranhão; MT: Mato Grosso; MS: Mato Grosso do Sul; MG: Minas Gerais; PA: Pará; PB: Paraíba; PR: Paraná; PE: Pernambuco; PI: Piauí; RJ: Rio de Janeiro; RN: Rio Grande do Norte; RS: Rio Grande do Sul; RO: Rondônia; RR: Roraima; SC: Santa Catarina; SP: São Paulo; SE: Sergipe; TO: Tocantins.

**Figure 4:**
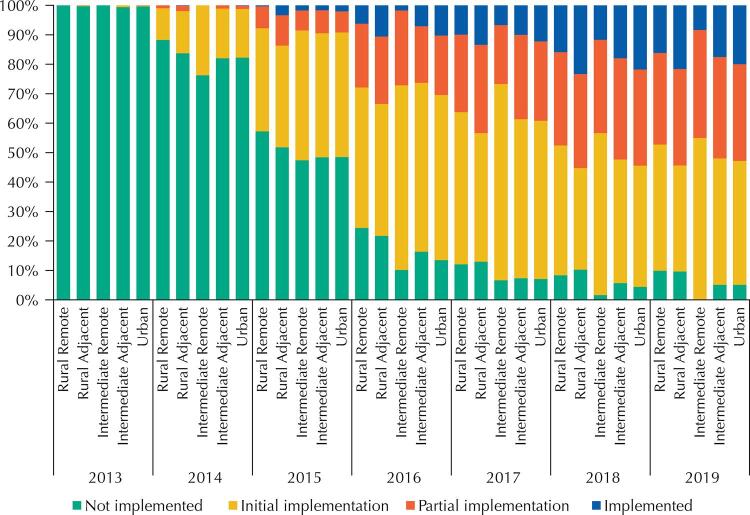
Implementation status of the e-SUS AB Strategy stratified by classification and characterization of rural and urban spaces in Brazil, 2013 to 2019.

Figure 4 shows the differences in relation to the classification and characterization of rural and urban spaces in Brazil. In 2019, the distribution of municipalities for each typology was ‘remote rural’ (322), ‘adjacent rural’ (3,022), ‘remote intermediate’ (60), ‘adjacent intermediate’ (680) and ‘urban’ (1,443). Municipalities classified as ‘adjacent rural’ and ‘urban’ had the highest percentage of implemented in all years of the study. In 2019, the highest percentage of ‘not implemented’ was from municipalities classified as ‘remote rural’ (9.9%).

## DISCUSSION

This is the first study conducted in Brazil to analyze the implementation of the e-SUS AB strategy. In addition, this study also considered the municipal sphere and the association between the characteristics of the municipalities and their performance in the strategy implementation process. The results mainly showed that, in 2019, 92.2% of Brazilian municipalities had a degree of e-SUS AB implementation distinct from ‘not implemented’. The South and Southeast regions stood out with higher percentages of strategy implementation, with the states with the highest percentages of ‘implemented’ status being Rio Grande do Sul (30.3%), São Paulo (29.5%), and Santa Catarina (28.8%).

Several factors and organizational arrangements affects the implementation of health information systems. Studies show that the degree of informatization, availability of internet connection, qualification and training of health professionals, and adequate IT (information technology) support^15,16^are key factors for the successful implementation of information systems. In addition, the characteristics of the system’s user interface may also influence the implementation process, including the functionality of the features and their usability, the quality of the data collected, and interoperability with other systems^17^.

A systematic review demonstrated that the process of implementing an information system is as important as the system itself. In this study, implementers’ concerns were patient privacy and safety, provider/patient relationship, staff anxiety, time required to implement the HIS, quality of care, financial issues, efficiency, and accountability^18^.

In Brazil, the e-SUS AB strategy was developed with support of the states and municipalities, represented by their entities, the *Conselho Nacional de Secretários de Saúde* (CONASS – National Council of Health Secretaries) and the *Conselho Nacional de Secretarias Municipais de Saúde* (CONASEMS – National Council of Municipal Health Secretaries). In the context of the results presented in this study, it is important to consider some previous actions of the Ministry of Health, developed since 2013, as preparatory to the implementation of the e-SUS AB strategy. Among these actions are the project *QualiSUS-Rede* (QualiSUS-Net), focused on the supply of equipment and peripherals for 486 municipalities; the support for the implementation for municipalities covered by 14 *Telessaúde* (Telehealth) centers; the training workshops for multipliers for municipalities with populations larger than 100 thousand inhabitants; the local support for municipalities via e-SUS AB consultants; the support for Dial 136 and for the Primary Care Department to solve doubts about the system and the supply of connectivity points for approximately 13 thousand Primary Care Units^11,19,20^.

In 2019, the Program to Support the Informatization and Qualification of Primary Health Care Data (*Informatiza APS*) was instituted by the Ministry of Health to computerize all Family Health Teams (eSF) and Primary Health Care Teams (eAP) in the country and to qualify the health data of the municipalities and the Federal District^21^, which certainly may have made an important contribution to the informatization scenario necessary for the implementation of the e-SUS AB strategy, especially in more vulnerable localities.

The results of this study showed that the e-SUS AB strategy has distinct moments of implementation, with the greatest degree of implementation at the beginning of the process, in 2013, in the Northeast and Southeast, while in subsequent years, the South region showed the greatest advance in implementation. This result may be influenced by the state of Rio Grande do Sul, which appears with one of the highest rates of implementation in recent years of the series studied, corroborating the results of a study that reported the experience of the participation of telehealth services to support the implementation, with better distribution of training in the local scenario, face-to-face and remote activities to support managers and professionals^20^.

The relationship between the degree of implementation and the type of municipality by the classification and characterization of rural and urban spaces in Brazil, made by IBGE, demonstrated the dependence between the variables. We saw lower implementation percentages in municipalities with the ‘remote rural’ and ‘remote intermediate’ typologies. Geographic and professional isolation may influence the implementation and require specific strategies to face the geographic barriers, such as the implementation of technologies of an interconnected communication network between health units and other levels of care that analyzes the peculiarities of the technological infrastructure, which are not always available in these locations^22^.

Another important aspect is the reliability of HIS. A systematic review evaluated studies conducted in Brazil and identified four priority dimensions of quality, which are reliability, validity, coverage and completeness^6^. The importance of good quality information indicates the need to establish a formal and regular evaluation policy for the HIS in Brazil, especially for those of national scope. This study did not directly address the quality of information provided in the e-SUS AB strategy, but the indicators used reflect, albeit in an exploratory way, some of the relevant aspects for information quality considerations.

In addition, computers and other technological resources are essential for the use of diagnostic and treatment systems, as well as information systems and electronic patient record systems that provide the information to support decision making. The better the computerized systems are able to record, store, and make available information, the better the information will be and the higher the quality in decision making will be^1,23^, with electronic medical record systems standing out.

Finally, the results of this study also add to the national discussion on monitoring and evaluation, in the scope of the *Política Nacional de Informação e Informática em Saúde* (PNIIS – National Health Information and Informatics Policy), establishing a series of guidelines that encourage, among other aspects, the promotion of strategies and mechanisms for the qualification of production and management of health information to strengthen e-Health, in the three spheres of SUS management^8^.

It is possible to understand the results of this study by considering its methodological limitations: first, other agents, in addition to health professionals, may have produced the records of the e-SUS AB strategy, since municipalities may adopt organizational arrangements where data entry and submission are performed outside the UBS, in administrative sectors of the health secretariats. On the other hand, there are municipalities using their own systems or systems marketed by third parties, where sending data in an adequate manner also depends on these service providers. Secondly, as the data in this study were not broken down at the sub-municipal level and in aggregate form, it is not possible to have an explicit vision of the flows of production and sending of the information analyzed.

Another important issue is that this study did not adopt a previous model validated by the literature to evaluate the degree of implementation of information systems, making it necessary to develop an original model for this analysis. Although not exactly a limitation, the discussion about the design adopted in this study to classify the implementation may benefit its validation and subsequent adoption, including in the health policy sphere, for monitoring and evaluation of this and other SUS strategies.

This study offers subsidies to support the public sector in monitoring and evaluating the implementation of information systems. It also presents useful solutions to improve the implementation of the e-SUS AB system, as well as contributing to the discussion about the model adopted in the provision of computerized systems, and to ground future studies in different areas of knowledge.

The results reinforce the need for consistent investments in the training of professionals to use the information system studied, systematic monitoring of the production of information – from collection to validation – and dissemination of data.
