# Supplementary material for: Implementation of the e-SUS Primary Care Strategy: an analysis based on official data
Source: Rev Saude Publica. 2022 Feb 23;56:5. doi: 10.11606/s1518-8787.2022056003405 (PMC8869366; doi:10.11606/s1518-8787.2022056003405)
Supplement: Translation

# Translation: Implantação da Estratégia e-SUS Atenção Básica: uma análise fundamentada em dados oficiais

**Authors:** Ana Claudia Cielo, Tainá Raiol, Everton Nunes da Silva, Jorge Otávio Maia Barreto

**Affiliations:** I Brasil Fundação Oswaldo Cruz. Programa de Mestrado Profissional em Políticas Públicas em Saúde. Brasília, DF, Brasil; II Brasil Universidade de Brasília. Faculdade de Ceilândia. Brasília, DF, Brasil

**Keywords:** Sistema Único de Saúde, Registros Eletrônicos de Saúde, Sistemas de Informação em Saúde, Atenção Primária à Saúde, Estratégias de eSaúde

## Abstract

**OBJETIVO:**

Analisar a implantação da estratégia e-SUS Atenção Básica (e-SUS AB) no Brasil entre os anos iniciais do sistema, de 2013 até 2019.

**MÉTODOS:**

Trata-se de um estudo quantitativo, descritivo e exploratório. Foram considerados os dados oficiais do Ministério da Saúde, enviados pelos municípios brasileiros, no período de abril de 2013 a dezembro de 2019. Os municípios foram categorizados como ‘não implantado’, ‘implantação inicial’, ‘implantação parcial’ e ‘implantado’, de acordo com os critérios definidos neste estudo. Verificou-se também se o tipo de município, segundo a classificação do IBGE, influenciou no grau de implantação da estratégia e-SUS AB. Foram realizadas análises descritivas e investigada a associação entre os graus de implantação do e-SUS AB e a tipologia da classificação e caracterização dos espaços rurais e urbanos do IBGE.

**RESULTADOS:**

O grau de implantação aumentou no período analisado. A situação de implantação da estratégia e-SUS AB, em 2019, foi ‘implantado’ em 20,2% (1.117) dos municípios, ‘implantação parcial’ em 32,9% (1.819), ‘implantação inicial’ 39,1% (2.159) e a situação ‘não implantado’ foi atribuída em 7,8% (432). As regiões Sul e Sudeste apresentaram a melhor situação de implantação em todos os anos e os estados do Rio Grande do Sul, São Paulo e Santa Catarina alcançaram um maior percentual de municípios com a situação ‘implantado’ em 2019.

**CONCLUSÕES:**

Houve avanço na implantação da estratégia e-SUS AB ao longo dos anos. A maior parte dos municípios encontra-se entre o status ‘implantação inicial’ e ‘implantação parcial’. Com isso, conclui-se que ainda são necessários investimentos em recursos tecnológicos, treinamento de profissionais e suporte para qualificar a implantação e uso de sistemas de informação no país, especialmente para a estratégia e-SUS AB.

## INTRODUÇÃO

Os sistemas de informação em saúde (SIS) são instrumentos padronizados de coleta de dados e monitoramento que têm como objetivo fornecer informações para análise em saúde, visando a melhoria do entendimento sobre os problemas de saúde da população^1,2^, subsidiando a tomada de decisão no âmbito das políticas públicas^3^.

Historicamente, os SIS brasileiros apresentam-se de forma fragmentada. Com múltiplas fontes, os dados coletados são consolidados com baixa qualidade e a sua disponibilização adota formato que dificulta sua apropriação e uso pelos gestores de saúde^4,5^. Desse modo, o monitoramento da qualidade dos dados que atendem ao Sistema Único de Saúde (SUS) não segue um plano regular de avaliações, havendo apenas iniciativas isoladas^6^.

O governo brasileiro, inspirado em experiências bem-sucedidas de países da Europa, Canadá, Austrália e Nova Zelândia, entre outros, concebeu e adotou a Estratégia e-Saúde para o Brasil. A Estratégia e-Saúde tem como objetivos aumentar a qualidade e ampliar o acesso à atenção à saúde, qualificar as equipes, agilizar o atendimento e melhorar o fluxo de informações para a tomada de decisão clínica, de vigilância, de regulação e de promoção da saúde, bem como a tomada de decisão voltada à gestão da saúde^7^, em consonância com a Política Nacional de Informação e Informática, que em sua última versão, de 2016, reforçou a importância de nortear as ações de tecnologia da informação e comunicação (TIC) e padronizar a obtenção e tratamento dos dados do sistema de saúde^8^.

No âmbito da atenção básica (AB), foi constituída a estratégia e-SUS Atenção Básica (e-SUS AB) no ano de 2013^9,10^, a qual se propunha oferecer um novo sistema de informação em saúde para atender às diferentes necessidades de informatização e organização dos municípios, visando à modernização da plataforma tecnológica, com apoio à gestão do cuidado, otimização da coleta de dados, interface com os diversos sistemas utilizados pela atenção básica e o aprimoramento do detalhamento das informações de saúde^11^, por meio do Cartão Nacional de Saúde, que permite a individualização dos registros, o que se apresentava como um grande desafio para o Brasil, pois rompia a lógica de dados consolidados utilizados na atenção básica até então.

Passados três anos da disponibilização do sistema, foi possível tornar obrigatório o envio de informações para a base de dados do Sistema de Informação em Saúde para a Atenção Básica (SISAB)^10^.

A estratégia e-SUS AB contempla o repositório nacional de informações SISAB e dois softwares de coleta para a entrada dos dados primários registrados pelos profissionais da AB: 1) Coleta de Dados Simplificada (CDS), por meio de fichas em papel e 2) Prontuário Eletrônico do Cidadão (PEC), que constitui um sistema informatizado. Além disso, ainda estão contemplados os sistemas comercializados por terceiros ou próprios já existentes nos municípios, integrados por meio de um mecanismo de importação de dados previamente definido^11^.

O movimento gerado pela mudança de sistema de informação impulsionou a informatização dos serviços de atenção básica em todo o país e pode ser considerado um caso de sucesso entre os sistemas disponibilizados em nível federal. Souza et al.^10^, destacam que, em 2018, a estratégia e-SUS AB estava presente nos 5.562 municípios brasileiros que possuem serviços de atenção básica e em torno de 98% das equipes de saúde da família (eSF), representando mais de 42,8 mil equipes.

Mesmo sendo visível o avanço, ainda são escassos os estudos que analisam a evolução da implantação do e-SUS AB, bem como dos fatores que influenciam esse processo e sobre o tempo necessário para a implantação da estratégia. É plausível que existam diferentes graus de implantação da estratégia, influenciados por características dos municípios, como sua localização, densidade populacional, urbanização, nível de informatização etc. Fatores geográficos e socioeconômicos, em geral, são relevantes para iniciativas governamentais que requerem a disponibilidade prévia de recursos específicos para sua implementação, ao passo que são mensuráreis e disponíveis, em bases de dados oficiais, facilitando seu uso para monitoramento dos avanços da implantação.

Este estudo analisou a implantação da estratégia e-SUS AB no Brasil no período de 2013 a 2019, investigando também características municipais que potencialmente influenciaram o melhor desempenho nesse processo.

## MÉTODOS

### Desenho do Estudo

Trata-se de um estudo quantitativo, descritivo e exploratório, com base em dados administrativos. O grau de implantação do e-SUS AB foi estimado em nível municipal, considerando o período de abril de 2013 a dezembro de 2019. Assim, a população do estudo consiste nos municípios brasileiros, que possuem oferta de serviços de AB registrados no Cadastro Nacional de Estabelecimentos de Saúde (CNES). O número de municípios apresentou variação nos anos estudados: 5.454 (2013); 5.496 (2014); 5.514 (2015); 5.517 (2016); 5.522 (2017); 5.524 (2018); e 5.527 (2019). O grau de implantação em cada município baseou-se nos dados fornecidos pelas equipes de atenção básica/saúde da família ao Ministério da Saúde referente ao uso do e-SUS AB. Foram excluídas deste estudo as equipes de Atenção Básica Prisional (EABP), Núcleo de Apoio a Saúde da Família (NASF), Consultório na Rua (CnR), Equipes de Saúde Bucal isolada (não vinculada a uma ESF) e Unidades Básicas de Saúde sem equipes vinculadas.

### Procedimentos Adotados para Estimar o Percentual de Implantação do e-SUS

As variáveis usadas neste estudo refletem o envio de dados de forma sistemática para o SISAB: competência (mês), Unidade Federada (UF), código de município do Instituto Brasileiro de Geografia e Estatística (IBGE), código de unidade de saúde do CNES, código de equipe do Identificador Nacional de Equipes (INE), código do tipo de equipe do CNES, categoria profissional e registros válidos na base nacional do CNES.

A análise dos dados foi precedida pela preparação da base de dados, que, por sua vez, compreendeu as seguintes etapas de tratamento: identificação das categorias profissionais ativas no CNES por mês; verificação do envio de informação para o SISAB das categorias profissionais ativas no CNES (médico, enfermeiro, técnico e auxiliar de enfermagem, dentista, técnico e auxiliar de saúde bucal e agente comunitário de saúde); análise do envio de informação por equipe de Atenção Básica/Saúde da Família para o SISAB. Nessa etapa, analisou-se se as categorias profissionais de cada equipe realizaram o envio de informação, classificando as equipes entre as que enviaram e as que não enviaram informações, de acordo com os critérios definidos para o estudo.

Na etapa seguinte, foram definidos e aplicados os critérios para caracterizar o grau de implantação do e-SUS AB, considerando a frequência e a regularidade do envio de dados para o SISAB. Primeiro, considerou-se envio dos dados por equipe: a) Envio Insuficiente – sem envio de informação ou envio de informação inferior a 30% das competências do ano; b) Envio Inicial – Envio de informação acima de 30% das competências do ano ou envio de informação por três (3) competências consecutivas no ano; c) Envio Parcial – Envio de informação acima de 50% das competências do ano e envio de informação por três (3) competências consecutivas no ano; d) Envio Satisfatório – Envio de informação acima de 80% das competências do ano e três (3) competências consecutivas no ano.

Em seguida, utilizou-se o resultado da etapa anterior para aferir o grau de implantação da estratégia e-SUS AB nos municípios: a) Não Implantado – mais de 80% das equipes do município classificadas como ‘envio insuficiente’ na fase anterior; b) Implantação Inicial – mais de 80% das equipes do município classificadas como ‘envio inicial’ ou entre 50% e 80% das equipes classificadas como ‘envio parcial’ ou entre 30% e 50% das equipes classificadas como ‘envio satisfatório’ na fase anterior; c) Implantação Parcial – mais de 80% das equipes do município classificadas como ‘envio parcial’ ou entre 50% e 80% das equipes classificadas como ‘envio satisfatório’ na fase anterior; d) Implantado – mais de 80% das equipes do município classificadas como ‘envio satisfatório’ na fase anterior. A Figura 1 apresenta o fluxo da classificação realizada.

**Figura 1:** Fluxograma da análise da implantação da Estratégia e-SUS AB.

### Análise Estatística

O cálculo dos dados foi realizado tendo como unidade de análise a ‘equipe’, sendo posteriormente agregado por município e finalmente por estado, por região geográfica e nacional. A apresentação dos dados se deu no formato de mapas e gráficos e a análise foi feita usando o a linguagem de programação *R* e *RStudio* (versão 1.1.463, 2009 – 2018^12^, pacotes plyr, dplyr, readxl e rlist).

Por fim, também foi usada a classificação de caracterização dos espaços rurais e urbanos do IBGE^13^, para investigar a implantação do e-SUS AB, considerando os grupos nela definidos. Essa classificação leva em conta aspectos relacionados à densidade populacional e sua distribuição no território dos municípios, para definir cinco grupos, aplicáveis à caracterização dos municípios brasileiros: urbano, intermediário adjacente, intermediário remoto, rural adjacente e rural remoto.

### Considerações Éticas

A pesquisa foi conduzida de acordo com os padrões éticos de pesquisa. Este estudo utilizou dados secundários e não identificados de sistemas de informação oficiais do Ministério da Saúde que está dispensado da apreciação do comitê de ética, conforme disposto na Resolução nº 510, de 7 de abril de 2016^14^.

## RESULTADOS

Os resultados agregados em escala nacional da implantação da estratégia e-SUS AB mostram crescimento expressivo na implantação do sistema. A evolução da implantação dessa estratégia no Brasil entre 2013 a 2019 está representada na Figura 2. Nos anos de 2013 e 2014, 99,7% e 83,4% dos municípios foram classificados com o status de ‘não implantado’, respectivamente. Em 2015, 49,2% dos municípios obtiveram algum grau de implantação diferente do status ‘não implantado’. Em 2016 e 2017, os status ‘implantação inicial’ e ‘implantação parcial’ ultrapassaram o percentual de ‘não implantado’ e a maior parcela encontrava-se entre os status ‘implantação inicial’, 49,7% e 48,3%, e ‘implantação parcial’, 21,6% e 28,7%, respectivamente. Nos dois últimos anos de análise, 2018 e 2019, 37,9% e 39,1%, dos municípios apresentavam o status ‘implantação inicial’, 32,4% e 32,9%, ‘implantação parcial’, e com status ‘implantado’, 21,7% e 20,2%, respectivamente.

**Figura 2:** Evolução da situação de implantação da Estratégia e-SUS AB por município, Brasil, de 2013 a 2019.

A evolução da implantação da estratégia e-SUS AB ocorreu de maneira distinta entre as regiões do país, conforme Figura 3. Em termos do percentual de implantação, as regiões Norte, Nordeste e Centro-Oeste ficaram abaixo da média nacional em todos os anos a partir de 2015, enquanto as regiões Sudeste e Sul ficaram acima da média nacional no mesmo período. No ano de 2019, os percentuais de municípios na situação ‘implantado’ e ‘implantação parcial’ superam a situação ‘não implantado’ e ‘implantação inicial’ no Brasil. Nesse aspecto, a região Sudeste apresenta a melhor situação, com 56,8% dos municípios nessa situação, seguida da região Sul, 54,1%, Norte, 53%, Nordeste, 51,4%, e Centro-Oeste, 44,5%. A análise de melhor percentual de municípios com status ‘implantado’ destaca a região Sul (24,8%), seguida da região Sudeste (24,4%), Centro-Oeste (16,9%), e Nordeste e Norte (15,4%), que apresentam o mesmo percentual.

**Figura 3:** Situação de implantação da Estratégia e-SUS AB por região geográfica, Brasil, de 2013 a 2019.

Na análise de implantação por Estado (Tabela 1), observou-se que o maior percentual de municípios com a situação ‘implantado’, em 2019, estava no estado do Rio Grande do Sul (30,3%), seguido de São Paulo (29,5%) e Santa Catarina (28,8%). O estado com situação mais deficitária é o Amapá, com 4,5% dos municípios com status ‘implantado’. O Distrito Federal, apesar de suas peculiaridades, não apresentou a situação ‘implantado’. Os estados brasileiros com maior percentual de municípios com a situação ‘não implantado’ foram Roraima com 20%, Paraná com 15,3%, Rio Grande do Sul e Goiás com 13,7%. O estado de Alagoas também não apresentou nenhum município com a situação ‘não implantado’.

**Tabela:** Implantação da Estratégia e-SUS AB por estado e região, 2013 a 2019.

Região/UF	Situação de implantação	2013 n (%)	2014 n (%)	2015 n (%)	2016 n (%)	2017 n (%)	2018 n (%)	2019 n (%)
Norte								
AC	Não implantado	22 (100)	18 (81,8)	10 (45,4)	6 (27,2)	3 (13,6)	0 (0)	2 (9)
	Implantação inicial	0 (0)	4 (18,1)	12 (54,5)	12 (54,5)	12 (54,5)	14 (63,6)	14 (63,6)
	Implantação parcial	0 (0)	0 (0)	0 (0)	4 (18,1)	7 (31,8)	7 (31,8)	5 (22,7)
	Implantado	0 (0)	0 (0)	0 (0)	0 (0)	0 (0)	1 (4,5)	1 (4,5)
AM	Não implantado	62 (100)	57 (91,9)	18 (29)	4 (6,4)	2 (3,2)	1 (1,6)	1 (1,6)
	Implantação inicial	0 (0)	5 (8)	41 (66,1)	43 (69,3)	41 (66,1)	30 (48,3)	30 (48,3)
	Implantação parcial	0 (0)	0 (0)	3 (4,8)	14 (22,5)	16 (25,8)	28 (45,1)	27 (43,5)
	Implantado	0 (0)	0 (0)	0 (0)	1 (1,6)	3 (4,8)	3 (4,8)	4 (6,4)
AP	Não implantado	16 (100)	13 (81,2)	7 (43,7)	8 (50)	6 (37,5)	3 (18,7)	1 (6,2)
	Implantação inicial	0 (0)	3 (18,7)	8 (50)	7 (43,7)	9 (56,2)	10 (62,5)	13 (81,2)
	Implantação parcial	0 (0)	0 (0)	1 (6,2)	1 (6,2)	1 (6,2)	3 (18,7)	2 (12,5)
PA	Não implantado	143 (100)	107 (74,3)	39 (27)	7 (4,8)	3 (2)	2 (1,3)	1 (0,6)
	Implantação inicial	0 (0)	37 (25,6)	84 (58,3)	91 (63,1)	95 (65,9)	74 (51,3)	73 (50,6)
	Implantação parcial	0 (0)	0 (0)	20 (13,8)	38 (26,3)	34 (23,6)	54 (37,5)	57 (39,5)
	Implantado	0 (0)	0 (0)	1 (0,6)	8 (5,5)	12 (8,3)	14 (9,7)	13 (9)
RO	Não implantado	52 (100)	47 (90,3)	26 (50)	6 (11,5)	3 (5,7)	2 (3,8)	1 (1,9)
	Implantação inicial	0 (0)	5 (9,6)	23 (44,2)	30 (57,6)	27 (51,9)	17 (32,6)	19 (36,5)
	Implantação parcial	0 (0)	0 (0)	3 (5,7)	13 (25)	18 (34,6)	24 (46,1)	19 (36,5)
	Implantado	0 (0)	0 (0)	0 (0)	3 (5,7)	4 (7,6)	9 (17,3)	13 (25)
RR	Não implantado	15 (100)	14 (93,3)	12 (80)	3 (20)	4 (26,6)	1 (6,6)	3 (20)
	Implantação inicial	0 (0)	1 (6,6)	3 (20)	12 (80)	8 (53,3)	10 (66,6)	8 (53,3)
	Implantação parcial	0 (0)	0 (0)	0 (0)	0 (0)	3 (20)	4 (26,6)	4 (26,6)
TO	Não implantado	139 (100)	111 (79,8)	84 (60,4)	36 (25,8)	19 (13,6)	15 (10,8)	13 (9,4)
	Implantação inicial	0 (0)	25 (17,9)	43 (30,9)	54 (38,8)	50 (35,9)	33 (23,9)	32 (23,1)
	Implantação parcial	0 (0)	3 (2,1)	10 (7,1)	35 (25,1)	48 (34,5)	45 (32,6)	55 (39,8)
	Implantado	0 (0)	0 (0)	2 (1,4)	14 (10)	22 (15,8)	45 (32,6)	38 (27,5)
Nordeste								
AL	Não implantado	102 (100)	98 (96)	58 (56,8)	2 (1,9)	0 (0)	0 (0)	0 (0)
	Implantação inicial	0 (0)	4 (3,9)	40 (39,2)	59 (57,8)	51 (50)	35 (34,3)	27 (26,4)
	Implantação parcial	0 (0)	0 (0)	4 (3,9)	36 (35,2)	36 (35,2)	41 (40,1)	48 (47)
	Implantado	0 (0)	0 (0)	0 (0)	5 (4,9)	15 (14,7)	26 (25,4)	27 (26,4)
BA	Não implantado	417 (100)	344 (82,4)	200 (47,9)	47 (11,2)	11 (2,6)	12 (2,8)	5 (1,1)
	Implantação inicial	0 (0)	72 (17,2)	187 (44,8)	297 (71,2)	267 (64)	190 (45,5)	190 (45,5)
	Implantação parcial	0 (0)	1 (0,2)	25 (5,9)	65 (15,5)	113 (27)	163 (39)	158 (37,8)
	Implantado	0 (0)	0 (0)	5 (1,1)	8 (1,9)	26 (6,2)	52 (12,4)	64 (15,3)
CE	Não implantado	184 (100)	183 (99,4)	92 (50)	22 (11,9)	7 (3,8)	7 (3,8)	5 (2,7)
	Implantação inicial	0 (0)	1 (0,5)	88 (47,8)	135 (73,3)	126 (68,4)	98 (53,2)	91 (49,4)
	Implantação parcial	0 (0)	0 (0)	4 (2,1)	21 (11,4)	36 (19,5)	62 (33,6)	66 (35,8)
	Implantado	0 (0)	0 (0)	0 (0)	6 (3,2)	15 (8,1)	17 (9,2)	22 (11,9)
MA	Não implantado	216 (99,5)	140 (64,5)	82 (37,7)	40 (18,4)	16 (7,3)	11 (5)	13 (5,9)
	Implantação inicial	1 (0,4)	74 (34,1)	118 (54,3)	138 (63,5)	149 (68,6)	125 (57,6)	119 (54,8)
	Implantação parcial	0 (0)	3 (1,3)	14 (6,4)	35 (16,1)	41 (18,8)	56 (25,8)	61 (28,1)
	Implantado	0 (0)	0 (0)	3 (1,3)	4 (1,8)	11 (5)	25 (11,5)	24 (11)
PB	Não implantado	220 (98,6)	114 (51,1)	43 (19,2)	22 (9,8)	10 (4,4)	10 (4,4)	10 (4,4)
	Implantação inicial	2 (0,8)	97 (43,4)	104 (46,6)	102 (45,7)	95 (42,6)	87 (39)	92 (41,2)
	Implantação parcial	1 (0,4)	9 (4)	58 (26)	73 (32,7)	85 (38,1)	75 (33,6)	84 (37,6)
	Implantado	0 (0)	3 (1,3)	18 (8)	26 (11,6)	33 (14,7)	51 (22,8)	37 (16,5)
PE	Não implantado	185 (100)	161 (87)	93 (50,2)	15 (8,1)	3 (1,6)	1 (0,5)	2 (1)
	Implantação inicial	0 (0)	23 (12,4)	83 (44,8)	131 (70,8)	116 (62,7)	94 (50,8)	86 (46,4)
	Implantação parcial	0 (0)	1 (0,5)	8 (4,3)	32 (17,2)	56 (30,2)	61 (32,9)	74 (40)
	Implantado	0 (0)	0 (0)	1 (0,5)	7 (3,7)	10 (5,4)	29 (15,6)	23 (12,4)
PI	Não implantado	224 (100)	212 (94,6)	138 (61,6)	62 (27,6)	24 (10,7)	18 (8)	20 (8,9)
	Implantação inicial	0 (0)	11 (4,9)	74 (33)	110 (49,1)	114 (50,8)	103 (45,9)	100 (44,6)
	Implantação parcial	0 (0)	1 (0,4)	10 (4,4)	45 (20)	70 (31,2)	70 (31,2)	71 (31,6)
	Implantado	0 (0)	0 (0)	2 (0,8)	7 (3,1)	16 (7,1)	33 (14,7)	33 (14,7)
RN	Não implantado	163 (97,6)	110 (65,8)	66 (39,5)	30 (17,9)	11 (6,5)	7 (4,1)	5 (2,9)
	Implantação inicial	4 (2,3)	48 (28,7)	75 (44,9)	93 (55,6)	82 (49,1)	81 (48,5)	76 (45,5)
	Implantação parcial	0 (0)	9 (5,3)	25 (14,9)	37 (22,1)	56 (33,5)	56 (33,5)	55 (32,9)
	Implantado	0 (0)	0 (0)	1 (0,5)	7 (4,1)	18 (10,7)	23 (13,7)	31 (18,5)
SE	Não implantado	75 (100)	45 (60)	31 (41,3)	10 (13,3)	1 (1,3)	4 (5,3)	4 (5,3)
	Implantação inicial	0 (0)	28 (37,3)	35 (46,6)	47 (62,6)	36 (48)	28 (37,3)	27 (36)
	Implantação parcial	0 (0)	2 (2,6)	7 (9,3)	13 (17,3)	33 (44)	32 (42,6)	29 (38,6)
	Implantado	0 (0)	0 (0)	2 (2,6)	5 (6,6)	5 (6,6)	11 (14,6)	15 (20)
Centro-oeste								
DF	Não implantado	1 (100)	1 (100)	1 (100)	1 (100)	1 (100)	0 (0)	0 (0)
	Implantação inicial	0 (0)	0 (0)	0 (0)	0 (0)	0 (0)	1 (100)	0 (0)
	Implantação parcial	0 (0)	0 (0)	0 (0)	0 (0)	0 (0)	0 (0)	1 (100)
	Implantado	0 (0)	0 (0)	0 (0)	0 (0)	0 (0)	0 (0)	0 (0)
GO	Não implantado	245 (99,5)	219 (89)	144 (58,5)	73 (29,6)	42 (17)	39 (15,8)	33 (13,4)
	Implantação inicial	1 (0,4)	25 (10,1)	78 (31,7)	113 (45,9)	126 (51,2)	108 (43,9)	105 (42,6)
	Implantação parcial	0 (0)	1 (0,4)	17 (6,9)	50 (20,3)	64 (26)	65 (26,4)	70 (28,4)
	Implantado	0 (0)	1 (0,4)	7 (2,8)	10 (4)	14 (5,6)	34 (13,8)	38 (15,4)
MS	Não implantado	79 (100)	63 (79,7)	44 (55,6)	15 (18,9)	6 (7,5)	5 (6,3)	3 (3,7)
	Implantação inicial	0 (0)	15 (18,9)	31 (39,2)	47 (59,4)	52 (65,8)	43 (54,4)	46 (58,2)
	Implantação parcial	0 (0)	1 (1,2)	3 (3,7)	15 (18,9)	17 (21,5)	23 (29,1)	16 (20,2)
	Implantado	0 (0)	0 (0)	1 (1,2)	2 (2,5)	4 (5)	8 (10,1)	14 (17,7)
MT	Não implantado	141 (100)	130 (92,1)	102 (72,3)	36 (25,5)	21 (14,8)	18 (12,7)	16 (11,3)
	Implantação inicial	0 (0)	10 (7)	32 (22,6)	75 (53,1)	76 (53,9)	51 (36,1)	56 (39,7)
	Implantação parcial	0 (0)	1 (0,7)	7 (4,9)	25 (17,7)	36 (25,5)	48 (34)	42 (29,7)
	Implantado	0 (0)	0 (0)	0 (0)	5 (3,5)	8 (5,6)	24 (17)	27 (19,1)
Sudeste								
ES	Não implantado	78 (100)	70 (89,7)	33 (42,3)	14 (17,9)	9 (11,5)	10 (12,8)	8 (10,2)
	Implantação inicial	0 (0)	7 (8,9)	39 (50)	47 (60,2)	51 (65,3)	42 (53,8)	48 (61,5)
	Implantação parcial	0 (0)	1 (1,2)	3 (3,8)	14 (17,9)	16 (20,5)	21 (26,9)	17 (21,7)
	Implantado	0 (0)	0 (0)	3 (3,8)	3 (3,8)	2 (2,5)	5 (6,4)	5 (6,4)
MG	Não implantado	850 (99,8)	741 (86,9)	486 (57)	128 (15)	76 (8,9)	59 (6,9)	63 (7,3)
	Implantação inicial	1 (0,1)	100 (11,7)	285 (33,4)	382 (44,8)	360 (42,2)	288 (33,7)	287 (33,6)
	Implantação parcial	0 (0)	11 (1,2)	70 (8,2)	210 (24,6)	275 (32,2)	280 (32,8)	295 (34,5)
	Implantado	0 (0)	0 (0)	11 (1,2)	132 (15,4)	142 (16,6)	226 (26,4)	208 (24,3)
RJ	Não implantado	91 (100)	68 (74,7)	36 (39,1)	5 (5,4)	7 (7,6)	3 (3,2)	7 (7,6)
	Implantação inicial	0 (0)	22 (24,1)	50 (54,3)	76 (82,6)	67 (72,8)	57 (61,9)	56 (60,8)
	Implantação parcial	0 (0)	1 (1)	5 (5,4)	8 (8,6)	16 (17,3)	25 (27,1)	23 (25)
	Implantado	0 (0)	0 (0)	1 (1)	3 (3,2)	2 (2,1)	7 (7,6)	6 (6,5)
SP	Não implantado	567 (99,1)	512 (86)	349 (57,7)	140 (23)	85 (13,9)	51 (8,3)	51 (8,3)
	Implantação inicial	4 (0,6)	71 (11,9)	182 (30,1)	226 (37,2)	218 (35,7)	167 (27,3)	187 (30,5)
	Implantação parcial	1 (0,1)	10 (1,6)	48 (7,9)	137 (22,5)	193 (31,6)	197 (32,2)	194 (31,6)
	Implantado	0 (0)	2 (0,3)	25 (4,1)	104 (17,1)	113 (18,5)	195 (31,9)	181 (29,5)
Sul								
PR	Não implantado	398 (100)	360 (90,2)	231 (57,8)	144 (36)	80 (20)	55 (13,8)	61 (15,3)
	Implantação inicial	0 (0)	37 (9,2)	134 (33,5)	166 (41,6)	200 (50,1)	135 (33,9)	170 (42,7)
	Implantação parcial	0 (0)	2 (0,5)	28 (7)	73 (18,2)	85 (21,3)	122 (30,6)	107 (26,8)
	Implantado	0 (0)	0 (0)	6 (1,5)	16 (4)	34 (8,5)	86 (21,6)	60 (15)
RS	Não implantado	459 (99,7)	360 (75,7)	175 (36,2)	103 (21,3)	94 (19,3)	73 (14,9)	67 (13,7)
	Implantação inicial	1 (0,2)	93 (19,5)	149 (30,8)	130 (26,9)	125 (25,7)	97 (19,8)	120 (24,5)
	Implantação parcial	0 (0)	22 (4,6)	105 (21,7)	135 (27,9)	146 (30,1)	139 (28,4)	153 (31,3)
	Implantado	0 (0)	0 (0)	54 (11,1)	115 (23,8)	120 (24,7)	179 (36,6)	148 (30,3)
SC	Não implantado	294 (100)	284 (96,2)	203 (68,8)	69 (23,3)	44 (14,9)	36 (12,2)	37 (12,5)
	Implantação inicial	0 (0)	11 (3,7)	75 (25,4)	117 (39,6)	114 (38,6)	75 (25,4)	87 (29,4)
	Implantação parcial	0 (0)	0 (0)	17 (5,7)	65 (22)	83 (28,1)	91 (30,8)	86 (29,1)
	Implantado	0 (0)	0 (0)	0 (0)	44 (14,9)	54 (18,3)	93 (31,5)	85 (28,8)
Total geral		5.454 (100)	5.496 (100)	5.514 (100)	5.517 (100)	5.522 (100)	5.524 (100)	5.527 (100)

UF: AC: Acre; AL: Alagoas; AP: Amapá; AM: Amazonas; BA: Bahia; CE: Ceará; DF: Distrito Federal; ES: Espírito Santo; GO: Goiás; MA: Maranhão; MT: Mato Grosso; MS: Mato Grosso do Sul; MG: Minas Gerais; PA: Pará; PB: Paraíba; PR: Paraná; PE: Pernambuco; PI: Piauí; RJ: Rio de Janeiro; RN: Rio Grande do Norte; RS: Rio Grande do Sul; RO: Rondônia; RR: Roraima; SC: Santa Catarina; SP: São Paulo; SE: Sergipe; TO: Tocantins.

A Figura 4 mostra as diferenças encontradas em relação à classificação e caracterização dos espaços rurais e urbanos do Brasil. No ano de 2019, a distribuição de municípios para cada tipologia era ‘rural remoto’ (322), ‘rural adjacente’ (3.022), ‘intermediário remoto’ (60), ‘intermediário adjacente’ (680) e ‘urbano’ (1.443). Os municípios com classificação ‘rural adjacente’ e ‘urbano’ foram os que apresentaram maior percentual de implantados em todos os anos do estudo. No ano de 2019, o maior percentual de ‘não implantado’ era dos municípios classificados como ‘rural remoto’ (9,9%).

**Figura 4:** Situação de implantação da Estratégia e-SUS AB estratificada pela classificação e caracterização dos espaços rurais e urbanos do Brasil, 2013 a 2019.

## DISCUSSÃO

Este é o primeiro estudo realizado no Brasil a analisar a implantação da estratégia e-SUS AB. Além disso, este estudo considerou também a esfera municipal e a associação entre as características dos municípios e seu desempenho no processo de implantação da estratégia. Os resultados mostraram principalmente que, em 2019, 92,2% dos municípios brasileiros apresentavam grau de implantação do e-SUS AB diferente de ‘não implantado’. As regiões Sul e Sudeste se destacaram com maiores percentuais de implantação da estratégia, sendo que os estados com maiores percentuais de status ‘implantado’ foram Rio Grande do Sul (30,3%), São Paulo (29,5%) e Santa Catarina (28,8%).

A implantação de sistemas de informação em saúde é condicionada por diversos fatores e arranjos organizativos. Estudos apontam que o grau de informatização, disponibilidade de conexão com a internet, qualificação e treinamento dos profissionais de saúde e suporte adequado de TI (tecnologia da informação)^15,16^ são fatores-chave para o sucesso da implantação de sistemas de informação. Além disso, o processo de implantação também pode ser influenciado pelas características da interface do sistema com o usuário, incluindo a funcionalidade dos recursos e sua usabilidade, a qualidade dos dados coletados e a interoperabilidade com outros sistemas^17^.

Uma revisão sistemática demonstrou que o processo de implantação de um sistema de informação é tão importante quanto o próprio sistema. Neste estudo, as preocupações dos implementadores foram a privacidade e a segurança dos pacientes, a relação fornecedor/paciente, a ansiedade da equipe, o tempo necessário para implementar o SIS, a qualidade do atendimento, as questões financeiras, a eficiência e a responsabilidade^18^.

No Brasil, a estratégia e-SUS AB foi desenvolvida com apoio dos estados e municípios, representados por suas entidades, o Conselho Nacional de Secretários de Saúde (CONASS) e o Conselho Nacional de Secretarias Municipais de Saúde (CONASEMS). No contexto dos resultados apresentados neste estudo, é importante registrar que algumas ações anteriores do Ministério da Saúde, desenvolvidas desde 2013, podem ser consideradas como preparatórias para a implantação da estratégia e-SUS AB. Entre essas ações estão o projeto QualiSUS-Rede, com enfoque na oferta de equipamentos e periféricos para 486 municípios; o apoio à implantação para municípios cobertos por 14 núcleos de Telessaúde; as oficinas de capacitação de multiplicadores para municípios com população maior que 100 mil habitantes; o apoio in loco para municípios por meio de consultores do e-SUS AB; o suporte do Disque 136 e do Departamento de Atenção Básica para solução de dúvidas sobre o sistema e a oferta de pontos de conectividade para aproximadamente 13 mil Unidades Básicas de Saúde^11,19,20^.

Já em 2019, o Programa de Apoio à Informatização e Qualificação dos Dados da Atenção Primária à Saúde (Informatiza APS) foi instituído pelo Ministério da Saúde para informatizar todas as equipes de Saúde da Família (eSF) e equipes de Atenção Primária à Saúde (eAP) do país e de qualificar os dados em saúde dos municípios e do Distrito Federal^21^, o que certamente pode ter contribuído de forma importante com o cenário de informatização necessário para a implantação da estratégia e-SUS AB, especialmente em localidades mais vulneráveis.

Os resultados deste estudo mostraram que a estratégia e-SUS AB tem momentos distintos de implantação, com maior grau de implantação no início do processo, em 2013, no Nordeste e no Sudeste, ao passo que, nos anos subsequentes, a região Sul mostrou o maior avanço de implantação. Esse resultado pode ter sido alavancado pelo estado do Rio Grande do Sul, que aparece com um dos maiores índices de implantação nos últimos anos da série estudada, corroborando os resultados de um estudo que relatou a experiência da participação de serviços de telessaúde para apoio à implantação, com capilarização dos treinamentos no cenário local, atividades presenciais e a distância de apoio a gestores e profissionais^20^.

A relação entre o grau de implantação e o tipo de município pela classificação e caracterização dos espaços rurais e urbanos do Brasil, feita pelo IBGE, demostrou a dependência entre as variáveis. Menores percentuais de implantação foram verificados em municípios com tipologia ‘rural remoto’ e ‘intermediário remoto’. O isolamento geográfico e profissional pode influenciar a implementação e requerer estratégias específicas para fazer frente às barreiras geográficas, como a implementação de tecnologias de uma rede interligada de comunicação entre unidades de saúde e outros níveis de atenção que analise as peculiaridades da infraestrutura tecnológica, que nem sempre estão disponíveis nesses locais^22^.

Outro aspecto a ser considerado é a fidedignidade dos SIS. Uma revisão sistemática avaliou estudos realizados no Brasil e identificou quatro dimensões prioritárias de qualidade, que são a confiabilidade, a validade, a cobertura e a completude^6^. A importância de informações com boa qualidade indica a necessidade de instituir no Brasil uma política de avaliação formal e regular dos SIS, especialmente para os de abrangência nacional. Este estudo não abordou diretamente a qualidade da informação provida na estratégia e-SUS AB, mas os indicadores utilizados refletem, ainda que de forma exploratória, alguns dos aspectos relevantes para considerações de qualidade da informação.

Ademais, computadores e outros recursos tecnológicos são essenciais para o uso de sistemas para elaboração de diagnósticos e tratamentos, bem como para a adoção de sistemas de informação e sistemas de prontuário eletrônico do paciente, que fornecem as informações para subsidiar o ato decisório. Quanto melhor os sistemas informatizados conseguem registrar, armazenar e disponibilizar informações, melhor será a informação e maior será a qualidade na tomada de decisão^1,23^, com destaque para os sistemas de prontuários eletrônicos.

Por fim, os resultados deste estudo também se agregam à discussão nacional sobre monitoramento e avaliação, no âmbito da Política Nacional de Informação e Informática em Saúde (PNIIS), estabelecendo uma série de diretrizes que incentivam, entre outros aspectos, a promoção de estratégias e mecanismos para a qualificação da produção e gestão da informação em saúde para fortalecer o e-Saúde, nas três esferas de gestão do SUS^8^.

Os resultados deste estudo devem ser interpretados considerando suas limitações metodológicas: em primeiro lugar, os registros realizados na estratégia e-SUS AB podem ter sido produzidos por outros agentes, além dos profissionais de saúde, dado que municípios podem adotar arranjos organizativos onde a digitação e envio dos dados são realizados fora das UBS, em setores administrativos das secretarias de saúde. Por outro lado, existem municípios que utilizam sistemas próprios ou comercializados por terceiros, onde o envio de dados de forma adequada depende também desses prestadores de serviços. Em segundo lugar, como os dados deste estudo não foram discriminados em nível submunicipal e de forma agregada, não é possível ter uma visão explícita sobre os fluxos de produção e envio da informação analisada.

Outra questão a ser considerada é que este estudo não adotou um modelo prévio validado pela literatura, para avaliação do grau de implantação de sistemas de informação, sendo necessário elaborar um modelo original para esta análise. Apesar de não ser exatamente uma limitação, a discussão sobre a modelagem adotada neste estudo para classificar a implantação, pode beneficiar sua validação e adoção posterior, inclusive no âmbito da política de saúde, para monitoramento e avaliação dessa e de outras estratégias do SUS.

Este estudo oferece subsídios para apoiar o setor público no monitoramento e avaliação da implantação de sistemas de informação. Também apresenta soluções úteis para melhorar a implantação do sistema e-SUS AB, além de contribuir para a discussão sobre o modelo adotado na disponibilização de sistemas informatizados, e de embasar estudos futuros nas diferentes áreas de conhecimento.

Os resultados reforçam a necessidade de investimentos consistentes em capacitação dos profissionais para o uso do sistema de informação estudado, acompanhamento sistemático da produção de informação – da coleta até a validação – e divulgação dos dados.
